# Impact of Depression, Fatigue, and Global Measure of Cortical Volume on Cognitive Impairment in Multiple Sclerosis

**DOI:** 10.1155/2015/519785

**Published:** 2015-03-11

**Authors:** Domenica Nunnari, Maria Cristina De Cola, Giangaetano D'Aleo, Carmela Rifici, Margherita Russo, Edoardo Sessa, Placido Bramanti, Silvia Marino

**Affiliations:** ^1^Neurobioimaging Laboratory, IRCCS Centro Neurolesi “Bonino Pulejo”, Via Provinciale Palermo, Contrada Casazza, 98124 Messina, Italy; ^2^Department of Biomedical Sciences and Morphological and Functional Imaging, University of Messina, Messina, Italy

## Abstract

*Objective*. To investigate the influence of demographic and clinical variables, such as depression, fatigue, and quantitative MRI marker on cognitive performances in a sample of patients affected by multiple sclerosis (MS). *Methods*. 60 MS patients (52 relapsing remitting and 8 primary progressive) underwent neuropsychological assessments using Rao's Brief Repeatable Battery of Neuropsychological Tests (BRB-N), the Beck Depression Inventory-second edition (BDI-II), and the Fatigue Severity Scale (FSS). We performed magnetic resonance imaging to all subjects using a 3 T scanner and obtained tissue-specific volumes (normalized brain volume and cortical brain volume). We used Student's *t*-test to compare depressed and nondepressed MS patients. Finally, we performed a multivariate regression analysis in order to assess possible predictors of patients' cognitive outcome among demographic and clinical variables. *Results*. 27.12% of the sample (16/59) was cognitively impaired, especially in tasks requiring attention and information processing speed. From between group comparison, we find that depressed patients had worse performances on BRB-N score, greater disability and disease duration, and brain volume decrease. According to multiple regression analysis, the BDI-II score was a significant predictor for most of the neuropsychological tests. *Conclusions*. Our findings suggest that the presence of depressive symptoms is an important determinant of cognitive performance in MS patients.

## 1. Introduction

More than 50% of patients with multiple sclerosis (MS) develop cognitive impairment in the course of the disease [1], even at the early stage [2]. The neuropsychological profile described in MS is characterized by complex attention, efficiency of information processing, executive functioning, processing speed, and long term memory [3]. Depressive symptoms are more common in MS patients than in the general population and in patients with other chronic medical conditions [4, 5]. Scientific evidence shows that depression is associated with cognitive functioning in MS patients [6, 7]. However, some other studies come to partially different conclusions. Karadayi et al. found no correlation between cognitive impairment and depression severity in 31 MS patients [8]. Borghi and colleagues showed that the best determinants of cognitive deficits in a large MS sample were the disease duration, the measure of disability, and the level of verbal intellectual functioning rather than psychiatric variables. However, the patients enrolled in this study had low levels of depression [9].

Since suicidal intent is common in patients with MS [10], the role of depressed mood in determining cognitive impairment is certainly still worthy of investigation. Fatigue is a clinical symptom often correlated with depression. In fact, they are two common syndromes in patients with MS, but their inherent relation and causes are still under debate.

A set of recent studies focused on neuropsychological and clinical variables omitting MR measurement [6, 9, 11–13]. Other works investigated the association of cognitive dysfunctions with cortical volume and clinical features in MS, but the assessment did not include a measure of depression [14] or fatigue [15].

The aim of the present cross-sectional study is to examine the interaction between mood disturbances, fatigue, values of normalized total and cortical volumes, disability, disease duration, and cognitive impairment in MS patients.

## 2. Material and Methods

### 2.1. Study Population

We enrolled 64 subjects (23 males and 41 females) with a diagnosis of MS according to the revised McDonald criteria [16] from the* IRCCS Centro Neurolesi “Bonino Pulejo”* of Messina, Italy. Subjects were consecutively recruited from June 2013 to February 2014 by expert neurologists of the MS center.

Exclusion criteria were as follows: >63 years of age, substance abuse, current corticosteroid use, history of serious psychiatric illness, and presence of neurological condition other than MS.

Four patients (3 males and 1 female) were excluded because of missing data. Thus, the study population consisted of 60 MS patients aged 21–61 years (39.35 ± 10.56) with a mean education level of 13.8 ± 3.8 years. 52/60 MS patients (86.7%) were classified as relapsing remitting MS and 8/60 (13.3%) as primary progressive. Subjects were clinically stable with no exacerbation or cortisone treatment within the last month. The mean disease duration was 6.3 ± 5.2 years and the mean Expanded Disability Severity Scale (EDSS) score was 2.4 ± 1.4. 55% (33/60) of the sample was pharmacologically treated: 22 patients with interferon, 3 patients with glatiramer acetate, 6 patients with natalizumab, and only one patient with fingolimod. One patient among these, treated with interferon, took also aminopyridine, whereas one subject had a psychotropic medication with SSRI. The remaining sample was not taking any drug during the study. The present study was approved by the Local Ethics Committee and written informed consent was obtained from all patients.  Table 1 shows demographic and clinical data.

### 2.2. Clinical and Neuropsychological Assessment

A trained psychologist, blinded to other clinical and MRI data, performed cognitive assessment of all patients using Rao's Brief Repeatable Battery of Neuropsychological Tests (BRB-N) [17]. This instrument is widely used in both clinical and research settings because of the high sensitivity and specificity in detecting cognitive dysfunctions in MS patients [18]. The BRB-N comprises seven tests, administered in approximately 30 minutes, in the following order:

- selective reminding test (SRT) to measure verbal learning (long term storage (LTS); consistent long term retrieval (CLTR));
- 10/36 spatial recall test (SPART) to measure visuospatial learning;
- symbol digit modality test (SDMT) to measure sustained attention and concentration;
- paced auditory serial addition test in two versions (PASAT-2 and PASAT-3) to measure information processing speed and working memory;
- delayed recall of the SRT (SRT-D) to assess retrieval from long term verbal memory;
- delayed recall of the SPART (SPART-D) to assess retrieval from long term visuospatial memory;
- word list generation (WLG) to measure semantic verbal fluency.

Performance on BRB-N was assessed by applying the available Italian normative values: a test was considered failed when the score was below the 5th percentile [19].

We detected depressive symptomatology with the Beck Depression Inventory-second edition (BDI-II), a self-report instrument recommended for MS patients [20] with 21 items rated on a scale from 0 to 3; the maximum total score was 63. We interpreted the BDI-II score according to manual guidelines [21]: minimal range = 0–13, mild depression = 14–19, moderate depression = 20–28, and severe depression = 29–63.

We assessed fatigue using the Fatigue Severity Scale (FSS) [22], a self-report questionnaire which consists of 9 items with a 7-point Likert scale. Finally, we measured patients' disability with the Expanded Disability Status Scale (EDSS) [23].

### 2.3. MR Examination

All patients and 25 sex-and-age-matched normal controls (NC) underwent a conventional and quantitative brain MRI on system operating at 3.0 T (Achieva, Philips, Netherlands) using a 32-channel SENSE head coil. We used a sagittal survey image to identify the anterior commissure and posterior commissure. We acquired a T1-weighted 3D fast field echo (FFE) sequence (TR 25 ms, TE 4.6 ms, flip angle 30°, FOV 240 × 240 mm^2^, matrix 256 × 256; voxel size 1 × 1 × 1 mm; slice thickness 1 mm) and a dual-echo, turbo spin-echo sequence (TR 25 ms, 256 × 256 matrix, FOV 250 × 250) yielding proton density-weighted and T2-weighted images in the transverse plane parallel to the line connecting the anterior commissure and posterior commissure.

We measured all cerebral volumes on T1-W 3D images by using the cross-sectional version of SIENA (structural image evaluation using normalization of brain atrophy) software, SIENAX (part of FSL 5.0: http://www.fmrib.ox.ac.uk/fsl/), a tool used to estimate the global brain volume normalized for head size. SIENAX registers the individual scan to the standard space brain and then converts the individual brain volume to a normalized brain volume and allows global measures of normalized brain volume (NBV) and the selective evaluation of normalized cortical volume (NCV).

### 2.4. Statistical Analysis

We performed statistical analyses using the 2.15.3 version of the open-source software R [24]. Since the Kolmogorov-Smirnov test results showed a normal distribution of the target variables, we performed a parametric analysis. We examined BDI-II, FSS, NBV, and NCV variables for outliers using Grubbs' tests. We expressed continuous variables in mean ± standard deviation and categorical variables in frequencies and percentages. We computed correlations between quantitative variables by Pearson's coefficient or by point-biserial correlation coefficient when one variable was dichotomous. We used Student's *t*-test (one- or two-tailed, where appropriate) to compare depressed patients (BDI-II ≥ 14) with nondepressed patients (BDI-II < 14). Finally, we performed a multivariate regression analysis to assess possible predictors of patients' cognitive outcome among demographic (age, gender, and education) and clinical (disease duration, EDSS, FSS, BDI-II, NBV, and NCV) variables. We applied a backward elimination stepwise procedure for the choice of the best predictive variables according to the Akaike information criterion (AIC). We considered a *P* value <0.05 as statistically significant.

## 3. Results

Table 1 summarizes the demographic and clinical characteristics of the MS patients. We did not find any statistically significant difference between men and women in either demographic or clinical variables. The sample's mean BDI-II score was 12.1 ± 6. Subdividing the patients according to the BDI-II cutoff, we observed a mild depression in 14 subjects (23.3%), a moderate depression in 7 subjects (11.7%), and a severe depression in only one subject (1.7%) who was detected as outlier by Grubbs' test. This patient's clinical picture was significantly different from the sample on BDI-II score (*t* = −34.08, *P* < 0.001), age (*t* = −9.39, *P* < 0.001), disease duration (*t* = 4.94, *P* < 0.001), NBV (*t* = −4.85, *P* < 0.001), and FSS (*t* = 5.43, *P* < 0.001). Thus, we decided to exclude her from further analysis.

### 3.1. Cognitive Assessment

**Figure 1:**
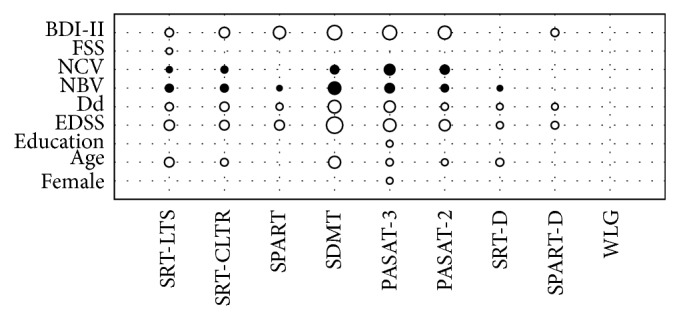
Significant correlations between the demographical and clinical variables and cognitive test scores. Positive correlations are in black and negative correlations in white. BDI-II, Beck Depression Inventory-II; FSS, Fatigue Severity Scale; NBV, normalized brain volume; NCV, normalized cortical volume; Dd, disease duration; EDSS, Expanded Disability Status Scale; SRT-LTS, selective reminding test-long term storage; SRT-CLTR, selective reminding test-consistent long term retrieval; SPART, 10/36 spatial recall test; SDMT, symbol digit modalities test; PASAT-3, paced auditory serial addition test, three-second interval version; PASAT-2, paced auditory serial addition test, two-second interval version; SRT-D, delayed recall of the selective reminding test; SPART-D, delayed recall of the 10/36 spatial recall test; WLG, word list generation.

At neuropsychological assessment, 27.12% of the sample (16/59) was cognitively impaired. We considered as cognitively impaired patients who failed in two or more cognitive tests. Overall, patients failed mainly on tasks concerning sustained attention and concentration (SDMT: 32.2%), information processing speed and working memory (PASAT-3: 30.5%), and visuospatial learning (SPART: 28.8%). Cognitive test scores had significant correlations with several clinical parameters (Figure 1). The main significant correlations are reported below.

Female gender and education weakly correlated only with PASAT-3 (*r* = 0.27, *P* < 0.05; *r* = −0.27, *P* < 0.05). Age was mainly related to SDMT (*r* = −0.40, *P* < 0.001), SRT-LTS (*r* = −0.39, *P* < 0.01), SRT-D (*r* = −0.32, *P* < 0.05), and SRT-CRTL scores (*r* = −0.31, *P* < 0.05). EDSS negatively correlated with SDMT (*r* = −0.66, *P* < 0.0001) and PASAT-3 (*r* = −0.51, *P* < 0.0001). Disease duration showed the same correlations pattern of EDSS, correlating mainly with SDMT (*r* = −0.51, *P* < 0.0001) and PASAT-3 (*r* = −0.46, *P* < 0.001). FSS resulted weakly correlated only with SRT-LTS (*r* = −0.26, *P* < 0.05). MR parameters, instead, positively correlated with SDMT (NBV: *r* = 0.55, *P* < 0.0001; NCV: *r* = 0.39, *P* < 0.01) and PASAT-3 (NBV: *r* = 0.43, *P* < 0.001; NCV: *r* = 0.48, *P* < 0.001). Finally BDI-II score was negatively related to SDMT (*r* = −0.58, *P* < 0.0001), PASAT (PASAT-3: *r* = −0.57, *P* < 0.0001; PASAT-2: *r* = −0.52, *P* < 0.0001), and SPART (*r* = −0.50, *P* < 0.0001).

### 3.2. Depression, Fatigue, and MRI Analysis

The mean FSS score in the sample was 19.9 ± 15.5. We found a moderate correlation between FSS and BDI-II scores (*r* = 0.40, *P* < 0.01), between FSS and EDSS scores (*r* = 0.29, *P* < 0.05), and between FSS scores and NBV (*r* = −0.26, *P* < 0.05).

NBV was 1490.7 ± 50.1 mm^3^ for patients and 1523.8 ± 22.5 mm^3^ for the NC (*P* < 0.01). The NCV was 540.0 ± 31.5 mm^3^ for patients and 578.0 ± 23.5 mm^3^ for the NC (*P* < 0.1).

We found a moderate correlation between these MR parameters and BDI-II (NBV: *r* = −0.41, *P* < 0.01; NCV: *r* = −0.39, *P* < 0.01). MR parameters were also strongly correlated with EDSS scores (*r* = −0.70, *P* < 0.0001 for NBC; *r* = −0.67, *P* < 0.0001 for NCV) and disease duration (NBV: *r* = −0.72, *P* < 0.0001; NCV: *r* = −0.69, *P* < 0.0001).

**Figure 2:**
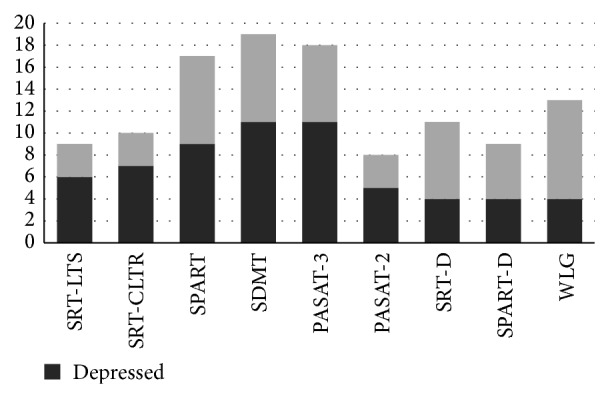
Depression in MS patients with cognitive deficit. BDI-II, Beck Depression Inventory-second edition; FSS, Fatigue Severity Scale; NCV, cortical normalized volume; NBV, normalized brain volume; Dd, disease duration; EDSS, Expanded Disability Severity Scale; SRT-LTS, selective reminding test-long term storage; SRT-CLTR, selective reminding test-consistent long term retrieval; SPART, 10/36 spatial recall test; SDMT, symbol digit modalities test; PASAT, paced auditory serial addition test; SRT-D, delayed recall of the selective reminding test; SPART-D, delayed recall of the 10/36 spatial recall test; WLG, word list generation.

In Table 2, we report the statistical comparisons between depressed and nondepressed patients. Although the two groups were not statistically different in age (*t* = 1.60, *P* > 0.05) and education (*t* = 0.35, *P* > 0.05), the FSS score was significantly higher and quantitative MRI markers were significantly lower in depressed than in nondepressed patients (FSS: *t* = 1.86, *P* < 0.05; NBV: *t* = −2.16, *P* < 0.05; NCV: *t* = −2.37, *P* < 0.05). We also found significant between group differences in most cognitive performances: depressed subjects achieved lower scores on SRT-CLTR (*t* = −2.94, *P* < 0.01), SPART (*t* = −3.64, *P* < 0.001), SDMT (*t* = −3.21, *P* < 0.01), PASAT-3 (*t* = −4.47, *P* < 0.0001), PASAT-2 (*t* = −3.79, *P* < 0.001), and SPART-D (*t* = −2.08, *P* < 0.05). On the other hand, patients with cognitive deficits were mainly depressed subjects (see Figure 2).

### 3.3. Multiple Regression Analysis

We performed multiple regression analysis to assess possible predictors of patients' cognitive outcome among demographic and clinical variables. The independent variables were age, disease duration, FSS, EDSS, BDI-II, NBV, and NCV. We report the estimates from a backward linear regression in Table 3. BDI-II was a significant predictor of better performance on most neuropsychological tests, including SRT-CLTR (*β* = −0.32, *P* = 0.01), SPART (*β* = −0.39, *P* < 0.01), SDMT (*β* = −0.30, *P* < 0.01), PASAT-3 (*β* = −0.45, *P* < 0.01), PASAT-2 (*β* = −0.43, *P* < 0.01), and SPART-D (*β* = −0.32, *P* = 0.01). Greater NCV predicted better cognitive performance on PASAT-3 (*β* = 0.25, *P* < 0.01) and PASAT-2 (*β* = 0.25, *P* = 0.04). EDSS score was a significant predictor of SRT-LTS (*β* = −0.30, *P* = 0.03) and SDMT (*β* = −0.44, *P* < 0.01) as well as age of SRT-D (*β* = −0.32, *P* = 0.01) and disease duration of SRT-CLTR (*β* = −0.26, *P* = 0.04).

## 4. Discussion

This study focused on the contribution of demographic and clinical variables, as the presence of depression and brain volume changes, to predict cognitive performance in a sample with MS. Our purpose was to extend the existing literature on multiple sclerosis by examining the relationship between depression, fatigue, neuropsychological performance, disease duration, disability, and values of normalized total and cortical volumes.

Confirming the literature [25], the cognitive tests our MS sample failed more likely were SDMT, PASAT, and SPART. Moreover, 36.7% of the subjects involved in the study were depressed. Considering the correlation analysis, in line with the literature [6, 26], we reported no relationship between the FSS score and BRB-N scores, except a weak correlation with SRT-LTS. Conversely, as explained above (Figure 2), the BDI score was strongly correlated with worse cognitive performances. Contrary to the results of some earlier studies [27], we find that the disease duration is negatively correlated with all test scores, except WLG.

Our MS sample had lower NBV and NCV values than the NC group.

Multiple regression analysis shows that, in our MS sample, verbal memory was affected by depression score, disability severity, age, and disease duration; visuospatial memory was affected by depression score; sustained attention and concentration were dependent on depression score and disability severity; and information processing speed was dependent on depression score and normalized cortical volume. It is important to note that the depression score is the most influential variable in terms of higher weight in regression models and cognitive domains affected. Overall, our findings suggest that the presence of depression is an important predictor of worse performance on cognitive tests in MS patients.

Depression in MS patients increases the suicide risk [28], cognitive dysfunctions [29], and the adherence to medication regimens [30]. Although the importance of addressing the occurrence of depression in MS patients can be inferred from this study, in our sample, only one subject was taking antidepressants, showing that patient's affective state may sometimes be overlooked. The Goldman Consensus Group's guidelines suggested the combination of SSRI pharmacotherapy and cognitive behavioral psychotherapy to treat depressive symptoms in MS (Goldman Consensus Group, 2005). Recent evidence highlights the effectiveness of cognitive behavioral therapy in treating depression in people with MS [31, 32].

A limitation of this study is the lack of evaluation of the lesion load. Cortical lesions are one of the factors with the potential to contribute to the development of cortical atrophy in patients with MS, which, in turn, may lead to more severe cognitive decline. However, to a visual assessment, our MS sample did not show cortical lesions in gray matter and we know that deep gray matter involvement could influence the development of cortical atrophy in patients with MS. In addition, not all studies on the development of cortical atrophy in patients with MS focused on brain lesions [33].

Despite the fact that several studies have shown the clinical relevance of cortical lesion assessment by means of DIR sequences, their use has some limitations: (a) DIR sequences are not widely available or standardized on most MRI scanners; (b) interobserver agreement is still low; (c) DIR imaging is susceptible to artifacts and has a poor sensitivity to detect subpial lesions, which are the most frequent and specific type of cortical lesions in patients with MS.

We note other limitations of this study: the sample size was relatively small and no follow-up evaluation was performed; the subgroup of PP patients was different from RR subjects with reference to a few clinical and demographic characteristics. However, despite these limitations, our results highlight the importance of considering the coexistence of mood disorders and clinical and MRI findings in the MS population.

In conclusion, the treatment of depressive symptoms is a clinician's duty in order to improve the quality of life of MS patients. Depression and fatigue are common symptoms of MS and they are the primary determinants of impaired quality of life in this disease. Untreated depression is associated with impaired cognitive function and poor adherence to treatment. For these reasons, systematic clinical and cognitive screening as well as management of depressive symptoms and fatigue should be recommended for all MS patients.

In this perspective, our study underscores the importance of early pharmacologic and rehabilitative intervention to manage cognitive changes in patients with MS.

## Figures and Tables

**Table 1:** Demographic and clinical characteristics of the MS patients.

	Females	Males	All
Patients	39 (65%)	21 (35%)	60 (100%)
Age (years)	38.6 ± 10.8	40.8 ± 10.3	39.3 ± 10.6
Education (years)	14.2 ± 3.8	12.9 ± 3.8	13.8 ± 3.8
Disease duration (years)	6.4 ± 5.3	6.1 ± 5.2	6.3 ± 5.2
EDSS (test scores)	2.2 ± 1.3	2.6 ± 1.6	2.4 ± 1.4
FSS (test scores)	19.5 ± 15.9	20.52 ± 15.2	19.9 ± 15.5
MR parameters			
Normalized brain volume (mm^3^)	1493.9 ± 43.3	1484.8 ± 55.7	1490.7 ± 50.1
Normalized cortical volume (mm^3^)	540.9 ± 31.2	538.4 ± 32.9	540.0 ± 31.5
MS subtype			
Relapsing remitting	35 (89.7%)	17 (81.0%)	52 (86.7%)
Progressive form	4 (10.3%)	4 (19.0%)	8 (13.3%)
Therapy			
None	15 (38.5%)	12 (57.1%)	27 (45.0%)
Interferon	17 (43.6%)	5 (23.8%)	22 (36.6%)
Glatiramer acetate	2 (5.1%)	1 (4.8%)	3 (5.0%)
Natalizumab	4 (10.3%)	2 (9.5%)	6 (10.0%)
Fingolimod	1 (2.5%)	—	1 (1.7%)
SNRI (sertraline)	—	1 (4.8%)	1 (1.7%)
Depression			
Minimal	24 (61.5%)	14 (66.7%)	38 (63.3%)
Mild	10 (25.6%)	4 (19.0%)	14 (23.3%)
Moderate	4 (10.3%)	3 (12.3%)	7 (11.7%)
Severe	1 (2.6%)	—	1 (1.7%)

Mean ± standard deviation was used to describe continuous variables; proportions (numbers and percentages) were used to describe categorical variables. EDSS, Expanded Disability Severity Scale; FSS, Fatigue Severity Scale; MR, magnetic resonance; SSRI, selective serotonin reuptake inhibitor.

**Table 2:** Statistical comparisons between depressed and nondepressed patients.

	Depressed Mean ± Std. dev	Nondepressed Mean ± Std. dev	One-tailed Student's *t*-test
Clinical features			
EDSS	2.9 ± 1.6	2.0 ± 1.2	***t* = 2.37, *P* = 0.02**
Dd	8.8 ± 5.6	5.0 ± 4.6	***t* = 2.65, *P* = 0.01**
NBV	1470.8 ± 53.6	1500.8 ± 45.8	***t* = −2.16, *P* = 0.02**
NCV	526.9 ± 33.5	547.5 ± 28.7	***t* = −2.37, *P* = 0.01**
FSS	25.3 ± 17.4	17.1 ± 13.9	***t* = 1.86, *P* = 0.02**

Neuropsychological assessment			
SRT-LTS	32.4 ± 14.5	38.1 ± 11.8	*t* = −1.54, *P* = 0.06
SRT-CLTR	20.9 ± 12.9	31.1 ± 12.7	***t* = −2.94, *P* < 0.01**
SPART	12.9 ± 3.7	16.9 ± 4.4	***t* = −3.64, *P* < 0.001**
SDMT	35.5 ± 14.8	47.8 ± 12.8	***t* = −3.21, *P* < 0.01**
PASAT-3	25.7 ± 10.3	38.1 ± 10.0	***t* = −4.47, *P* < 0.001**
PASAT-2	21.1 ± 8.2	29.4 ± 7.8	***t* = −3.79, *P* < 0.001**
SRT-D	6.9 ± 2.8	7.4 ± 2.3	*t* = −0.53, *P* = 0.30
SPART-D	5.0 ± 1.8	6.1 ± 2.1	***t* = −2.08, *P* = 0.02**
WLG	20.0 ± 4.9	20.8 ± 6.2	*t* = −0.57, *P* = 0.29

EDSS, Expanded Disability Severity Scale; Dd, disease duration; NBV, normalized brain volume; NCV, cortical normalized volume; FSS, Fatigue Severity Scale; SRT-LTS, selective reminding test-long term storage; SRT-CLTR, selective reminding test-consistent long term retrieval; SPART, 10/36 spatial recall test; SDMT, symbol digit modalities test; PASAT, paced auditory serial addition test; SRT-D, delayed recall of the selective reminding test; SPART-D, delayed recall of the 10/36 spatial recall test; WLG, word list generation.

Statistically significant differences are in bold.

**Table 3:** Backward linear regression: significant predictors of performance on each neuropsychological test.

Dependent variables	Predictors	*β*	Std *β*	*P* value	Adjusted *R* ^2^
SRT-LTS	EDSS	−2.74	−0.30	0.03	0.19
SRT-CLTR	Dd BDI-II	−0.67 −0.83	−0.26 −0.32	0.04 0.01	0.20
SPART	BDI-II	−0.34	−0.39	<0.01	0.25
SDMT	EDSS BDI-II	−4.55 −0.83	−0.44 −0.30	<0.01 <0.01	0.52
PASAT-3	BDI-II NCV	−0.99 0.11	−0.45 0.25	<0.01 <0.01	0.37
PASAT-2	BDI-II NCV	−0.72 0.07	−0.43 0.25	<0.01 0.04	0.30
SRT-D	Age	−0.08	−0.32	0.01	0.09
SPART-D	BDI-II	−0.13	−0.32	0.01	0.09
WLG	NCV	−0.07	−0.41	0.03	0.09

*β*, regression coefficient; Std *β*, standardized regression coefficient; SRT-LTS, selective reminding test-long term storage; SRT-CLTR, selective reminding test-consistent long term retrieval; SPART, 10/36 spatial recall test; SDMT, symbol digit modalities test; PASAT, paced auditory serial addition test; SRT-D, delayed recall of the selective reminding test; SPART-D, delayed recall of the 10/36 spatial recall test; WLG, word list generation; EDSS, Expanded Disability Severity Scale; Dd, disease duration; BDI-II, Beck Depression Inventory-second edition; NCV, normalized cortical volume.
